# A New Heteroleptic Zn(II) Complex with Schiff Bases Sensitizes Triple-Negative Breast Cancer Cells to Doxorubicin and Paclitaxel

**DOI:** 10.3390/pharmaceutics16121610

**Published:** 2024-12-18

**Authors:** Raiane Aparecida dos Santos Machado, Raoni Pais Siqueira, Fernanda Cardoso da Silva, André Carlos Pereira de Matos, Dayanne Silva Borges, Gislaine Gonçalves Rocha, Thais Cristina Prado de Souza, Rafael Aparecido Carvalho Souza, Clayton Rodrigues de Oliveira, Antônio G. Ferreira, Pedro Ivo da Silva Maia, Victor Marcelo Deflon, Carolina Gonçalves Oliveira, Thaise Gonçalves Araújo

**Affiliations:** 1 Laboratory of Genetics and Biotechnology, Institute of Biotechnology, Federal University of Uberlândia, Patos de Minas 38700-002, MG, Brazil; machado.raiane@hotmail.com (R.A.d.S.M.); raoni.siqueira@ufu.br (R.P.S.); fernanda.cardoso95@yahoo.com (F.C.d.S.); andre.carlos@ufu.br (A.C.P.d.M.); daaysborges@gmail.com (D.S.B.); gislaineg.rocha08@gmail.com (G.G.R.); 2 Institute of Chemistry, Federal University of Uberlândia, Uberlândia 38400-902, MG, Brazil; psthaiscristina@gmail.com (T.C.P.d.S.); rafasouza27@hotmail.com (R.A.C.S.); 3 Department of Chemistry, Federal University of São Carlos, Rodovia Washington Luís, km 235, São Carlos 13565-905, SP, Brazil; claytonrol@hotmail.com (C.R.d.O.); giba@ufscar.br (A.G.F.); 4 Bioactive Compounds Development Research Group, Federal University of Triangulo Mineiro, Av. Dr. Randolfo Borges 1400, Uberaba 38025-440, MG, Brazil; pedro.maia@uftm.edu.br; 5 São Carlos Institute of Chemistry, University of São Paulo, São Carlos 13560-970, SP, Brazil; deflon@iqsc.usp.br; 6 Laboratory of Nanobiotechnology Prof. Dr. Luiz Ricardo Goulart Filho, Institute of Biotechnoloy, Federal University of Uberlândia, Uberlandia 38405-302, MG, Brazil

**Keywords:** Zinc(II), MDA-MB-231, breast cancer, chemotherapy

## Abstract

**Background/Objectives**: Triple-negative breast cancer (TNBC) is the most challenging molecular subtype of breast cancer (BC) in clinical practice, associated with a worse prognosis due to limited treatment strategies and its insensitivity to conventional drugs. Zinc is an important trace element for homeostasis, and its Schiff base metal complexes have shown promise in treating advanced tumors. In this study, four new heteroleptic Zn(II) complexes (**1**–**4**) with Schiff bases were synthesized, characterized, and evaluated for their activity in BC cells. **Methods**: Compounds were synthesized, characterized, and their crystal structures were determined. Biological activity was assessed using MTT, clonogenic, scratch wound healing, caspase 3 and 8 activity, qPCR, and chemosensitization assays. **Results**: The complexes exhibited cytotoxicity against MCF-7 (luminal BC), MDA-MB-453 (HER2-positive BC), and MDA-MB-231 (TNBC) cell lines, with IC_50_ values ranging from 0.01 to 20 µM. Complex **4** showed reduced cytotoxicity toward non-tumor cell lines. This, complexation with Zn(II) increased the cytotoxicity of the ligands, a trend not observed for complexes **1**–**3**. Due to its favorable profile, complex **4** was selected for further assays, in which it inhibited colony formation and the cell migration of TNBC cells in a dose-dependent manner. Furthermore, this compound induced cell death independently of caspases, decreasing the activity of caspase 8. Interestingly, complex **4** sensitized TBNC cells to doxorubicin and paclitaxel, possibly modulating the epithelial–mesenchymal transition mechanism, as evidenced by increased *CDH1* expression. **Conclusions**: Results suggest the potential of complex **4** in sensitizing aggressive BC cells to chemotherapy, proving to be a promising alternative in cases of therapeutic failure.

## 1. Introduction

Cancer is one of the leading causes of death worldwide, representing a significant barrier to increasing life expectancy in all countries. In 2022, breast cancer (BC) was responsible for 670,000 deaths worldwide, with it being the most common cancer among women in 157 countries [1]. The incidence of BC has an increasing profile, and epidemiological data have been severely impacted by the COVID-19 pandemic, leading many women to delay healthcare treatment, which suggests the occurrence of severe cases of the disease in the coming years [2].

BC is a heterogeneous disease characterized by a complex array of molecular alterations that drive its development and progression. In this context, breast tumors are classified molecularly, which has guided clinical management toward biology-centered approaches. Currently, five subtypes are adopted, with those expressing an estrogen receptor (ER) and/or progesterone receptor (PR) classified as hormone receptor-positive BCs. In contrast, triple-negative breast cancer (TNBC) is defined by its lack of expression of an ER, PR, and human epidermal growth factor receptor 2 (HER2) [3]. Unfortunately, therapeutic strategies for triple-negative tumors are limited and still focused on surgery, chemotherapy, and radiotherapy. Although TNBC often exhibits a higher pathological complete response (pCR) rate to taxanes, anthracycline-based, or platinum-based systemic therapies, these tumors are typically poorly differentiated, highly aggressive, and associated with the worst prognosis [4]. About 46% of patients present distant metastases, mainly in the third year after diagnosis. In these cases, the tumor can reach the brain and visceral organs, with a mortality rate of 75% within three months after recurrence [5]. Therapeutic resistance exacerbates this scenario, as 50% of patients with TNBC do not respond to standard drugs and progress to a the disease becoming fatal [6]. Therefore, the development of new treatment strategies is crucial, particularly to improve these patients’ prognoses and quality of life.

Zinc (Zn) is an essential trace element that regulates normal cellular activity, acting as a cofactor for more than 300 enzymes involved in gene expression, enzymatic activity, and protein structure [7]. Consequently, various cellular mechanisms are responsive to Zn homeostasis, including apoptosis, autophagy, metabolism, cell proliferation and differentiation, immune response, and cell signaling [8]. In BC, Chandler and colleagues [9] noted abnormal Zn levels associated with molecular subtypes, grade, invasiveness, metastatic potential, and response to therapy. Although the results are inconsistent, elevated Zn levels have been documented in tissues from patients with BC [10]. Therefore, the demand for Zn by tumor cells presents a new therapeutic avenue, wherein structural designs and different ligands can be explored. Zn(II) ions facilitate influx into tumor cells and possess Lewis acidity, favorable redox characteristics, easy ligand exchange, adaptable coordination environments, and substantial physiological availability [11].

The chemistry of coordination compounds has garnered significant attention in oncology, with novel metal centers and ligands being investigated to achieve desired biological functions. Complexes containing Zn(II) have shown potential cytotoxicity against lung (A549) [12], ovarian (HeLa), and breast (MCF-7) cancer cell lines, including TNBC (MDA-MB-231, MDA-MB-468, HCC1937, and Hs 578T) [13]. Schiff bases are organic chelates synthesized through the condensation of a carbonyl group with a primary amine, exhibiting anti-inflammatory and antitumor activities [14]. In previous studies, Schiff base Zn(II) complexes were synthesized and demonstrated cytotoxic, cytostatic, and genotoxic effects on mammary tumor cells, including the MCF7, T47-D, and MDA-MB-231 BC cell lines [15,16]. Additionally, Zn(II) complexes containing thiosemicarbazones, such as [Zn(atc-R)2] (where atc = acetylpiridyne-N(4)-R-thiosemicarbazone and R = ethyl or phenyl), exhibited promising selectivity indices against the MDA-MB-231 cell line [17].

Herein, we evaluated the in vitro effects of four novel Zn(II) mixed-ligand complexes, derived from two different ligands belonging to the semicarbazone, hydrazone, and thiosemicarbazone classes. We also investigated the impact of these complexes on cell viability, gene expression, and the sensitivity of TNBC cells to commonly used chemotherapeutics.

## 2. Materials and Methods

### 2.1. Chemicals, Drugs, and Reagents

Dimethyl sulfoxide (DMSO) was purchased from Sigma-Aldrich^®^ (Saint Louis, MO, USA). 3–4,5 dimethylthiazol-2, 5 diphenyl tetrazolium bromide (MTT) was purchased from Life Technologies^®^. Vitamin D (VD) was purchased from Purifarma^®^. Paclitaxel and Doxorubicin were purchased from Sigma-Aldrich^®^. Crystal Violet was purchased from ACS Científica^®^. Methanol and Acetic Acid were purchased from Vetec^®^ (Rio de Janeiro, RJ, Brazil).

2-Acetylpyridine, 4-ethyl-3-thiosemicarbazide, semicarbazide, zinc chloride and analytical reagent-grade chemicals and solvents were obtained commercially and used without further purification. The 4-cyclohexyl-3-thiosemicarbazide was prepared as previously described [18]. The ligands Hsc, Hatc-Et, Hatc-Ch, and Hhz were prepared by refluxing equimolar ethanolic solutions containing the desired hydrazide (10 mmol) and 2-acetylpyridine (10 mmol) for 1 h, as reported elsewhere [19].

### 2.2. Synthesis of the Compounds

In this work, four novel heteroleptic Zn(II) complexes, [Zn(Hz)(atc–Et)] (**1**), [Zn(atc–Et)(atc–Ch)] (**2**), [Zn(atc–Et)(Hsc)]Cl (**3**), and [Zn(Hz)(Hsc)]Cl (**4**), were prepared. The mixed-ligand Zn(II) complexes were synthesized by using the pentacoordinate complex [ZnCl_2_(HL)] as a starting material as reported in the literature [20,21,22]. Briefly, 0.1 mmol of ZnCl_2_ was added to a solution containing the desired ligands (0.1 mmol) in 30 mL of EtOH. After the formation of the white precipitates, the solids were filtered off and dried. The heteroleptic Zn(II) complexes were synthesized by using equimolar amounts of the desired ligand and [ZnCl_2_(HL)] (1:1) in 15 mL of MeCN followed by the addition of Et_3_N. The mixture was heated at reflux for 4 h. The yellow solids obtained were filtered off, washed with methanol and *n*-hexane, and dried under a vacuum.

[Zn(atc-Et)(hz)] (1). MM (g/mol): 514.91. Yield: 63%. Color: yellow. Anal. Calc. for C_22_H_23_N_7_O_2_SZn: C, 51.32; H, 4.50; N, 19.04; O, 6.21; S, 6.23%. Found: C, 51.16; H, 4.62; N, 19.38; O, 6.52; S, 5.98. IR (ν_max_/cm^−1^): 3253 ν(N-H), 1593, 1581 ν(C=N) + ν(C=C), 761 ν(C-S), 1228 ν(CO). Molar conductivity (1 × 10^−3^ mol L^−1^ DMSO): 0.24 μS/cm. ^1^H NMR (400 MHz, DMSO-*d*_6_) δ 7.95–7.72 (m, 7H, Fu + Py + NH), 7.39–7.27 (m, 3H, Py), 7.01–6.97 (m, 1H, Py), 6.57–6.53 (m, 1H, Py), 2.72–2.53 (m, 8H, CH_3_ CH_2_CH_3_), 1.14 (s, 3H, CH_3_).

[Zn(atc-Et)(atc-Ch)] (2): MM (g/mol): 562.08. Yield: 54%. Color: yellow. Anal. Calc. for C_24_H_32_N_8_S_2_Zn: C, 51.29; H, 5,74; N, 19.94; S, 11.41%. Found: C, 51.16; H, 5.81; N, 19.61; S, 11.63. IR (ν_max_/cm^−1^): 3248 ν(N-H), 1591, 1550 ν(C=N) + ν(C=C), 1074 ν(N-N), 779 ν(C-S). Molar conductivity (1 × 10^−3^ mol L^−1^ DMSO): 0.35 μS/cm. ^1^H NMR (400 MHz, DMSO-*d*_6_) δ 7.85 (t, *J* = 7.8 Hz, 2H, NH), 7.79–7.68 (m, 5H, Py), 7.29–7.22 (m, 3H, Py), 3.34 (s, 2H, Cy), 2.57 (s, 7H, CH_2_,CH_3_, Cy), 1.95 (s, 2H, Cy), 1.79–1.49 (m, 3H, Cy), 1.24 (t, *J* = 7.8 Hz, 3H, CH_3_), 1.14 (t, *J* = 6.9 Hz, 5H, CH_3_, Cy).

[Zn(Hsc)(atc-Et)]Cl (3): MM (g/mol): 500.33. Yield: 60%. Color: yellow. Anal. Calc. for C_18_H_23_ClN_8_OSZn: C, 43.21; H, 4.63; N, 22.40; O, 3.20; S, 6.41%. Found: C, 43.41; H, 4.77; N, 22.21; O, 3.41; S, 6.28. IR (ν_max_/cm^−1^): 3365, 3299, 3252, 3175 ν(N-H), 1600, 1520 ν(C=N) + ν(C=C), 785 ν(C-S), 1678 ν(C=O). Molar conductivity (1 × 10^−3^ mol L^−1^ methanol): 100 μS/cm. ^1^H NMR (400 MHz, DMSO-*d*_6_) δ 8.69–8.19 (m, 2H, Py), 8.07 (t, *J* = 7.5 Hz, 1H, NH), 7.80 (m, 3H, Py), 7.60–7.21 (m, 3H, Py), 6.62 (s, 2H, NH_2_), 2.57 (s, 2H, CH_2_), 2.47 (s, 3H, CH_3_), 2.30 (s, 3H, CH_3_), 1.14 (d, *J* = 14.3 Hz, 3H, CH_3_).

[Zn(Hsc)(hz)]Cl (4): MM (g/mol): 507.26. Yield: 48%. Color: yellow. Anal. Calc. for C_22_H_22_N_8_O_3_Zn: C, 51.62; H, 4.33; N, 21.89; O, 9.65%. Found: C, 51.42; H, 4.41; N, 21.84; O, 9.38. IR (ν_max_/cm^−1^): 3427, 3304, 3240 ν(NH), 1643 ν(C=O), 1595 ν(C=N) + ν(C=C), 1190 ν(C-O), 1683 ν(C=O). Molar conductivity (1 × 10^−3^ mol L^−1^ methanol): 95 µS cm^−1^. ^1^H NMR (400 MHz, DMSO-*d*_6_) δ 8.53 (s, 2H, NH_2_), 8.13 (t, *J* = 8.2 Hz, 2H, Fu), 8.05–7.92 (m, 4H, Py), 7.87 (d, *J* = 5.6 Hz, 1H, Fu), 7.77–7.73 (m, 1H, Py), 7.62 (s, 1H, NH), 7.40 (ddd, *J* = 7.6, 5.0, 1.2 Hz, 1H, Py), 7.11–6.98 (m, 2H, Py), 2.70 (s, 3H, CH_3_), 2.59 (s, 3H, CH_3_).

### 2.3. Physical Measurements

FTIR spectra were measured as KBr pellets on a Shimadzu IR Prestige-21 spectrophotometer between 400 and 4000 cm^−1^, with a resolution of 4 cm^−1^. The collection of the spectra was performed with 32 scans and the data were treated in Schimadzu IRsolution 1.40 software. Elemental analyses were determined using a Perkin Elmer (Waltham, MA, USA) 2400 CHNS/O Analyzer. The conductivities of the complexes were measured in MeOH solutions using an Orion Star Series conductometer. ^1^H NMR measurements were recorded at 14.1 T on a Bruker (Billerica, MA, USA) Avance 600 spectrometer. COSY spectra were performed at 298 K on a Bruker DRX 400 MHz spectrometer, at 9.4 T.

### 2.4. Crystal Structure Determination

Yellow crystals were grown through the slow evaporation of the mother solution of complex **3** at room temperature. The data collection was performed using Mo-K_α_ radiation (λ = 0.71073 Å) on a BRUKER APEX II Duo diffractometer. Multi-scan absorption correction was carried out for **3** by the SADABS program [23]. The structures were solved with SHELXS97 [24] or SHELXT [25] and all non-hydrogen atoms were refined with anisotropic displacement parameters with SHELXL2016 [26], programs included in the OLEX2 program package [27]. The hydrogen atoms were calculated at idealized positions using the riding model option of SHELXL2016 [26]. Structural representation was drawn using ORTEP-3 [28] and MERCURY [29]. The structures’ data have been deposited in the Cambridge Crystallographic Data center under accession numbers 2379146 (for complex **3**) and 2407222 (for complex **1**). Table S3 presents more detailed information about the crystal structure determination and refinement data.

### 2.5. Cell Culture

For in vitro assays, three tumor cell lines were used: MCF7 (ER-positive BC), maintained in Roswell Park Memorial Institute medium (RPMI-1640, Gibco^®^, Grand Island, NY, USA); MDA-MB-453 (HER2-positive BC), grown in Iscove’s Modified Dulbecco’s Medium (IMDM, Gibco^®^); and MDA-MB-231 (TNBC), grown in Leibovitz’s L-15 Medium (L15, Gibco^®^). Additionally, three non-tumor cell lines were used: HUVEC (human umbilical vein endothelial cells), cultured in (RPMI-1640); HFF (human fibroblast), cultured in Dulbecco’s Modified Eagle Medium: Nutrient Mixture—High Glucose (DMEM/F12-High Glucose, Gibco^®^); and MCF10A (human mammary epithelial cell line), also cultured in DMEM/F12 supplemented with 10 μg/mL of insulin (Gibco^®^), 0.25 μg/mL of hydrocortisone (Sigma-Aldrich^®^, Saint Louis, MO, USA), and 10 μg/mL of Epidermal Growth Factor (EGF) (Gibco^®^). All culture media were supplemented with 10% fetal bovine serum (FBS) (Gibco^®^) and 50 μg/mL of gentamicin (Cultilab^®^, Campinas, SP, Brazil). The culture flasks were kept in an incubator at 37 °C under an atmosphere of 5% CO_2_, except for the MDA-MB 231 lineage, which was grown without CO_2_ gas exchange. All cells were purchased from the American Type Culture Collection (ATCC, Manassas, VA, USA).

### 2.6. MTT Assay

To analyze cell viability, the test based on the reduction of tetrazolium 3-(4,5-dimethylthiazol-2yl)-2,5-diphenyl bromide (MTT) was used. HUVEC cell lines (1.0 × 10^4^ cells/well); HFF (8.0 × 10^3^ cells/well); MCF10A (1.2 × 10^4^ cells/well); MDA-MB-453 and MCF7 (5.0 × 10^3^ cells/well) and MDA-MB-231 (1.5 × 10^4^ cells/well) were seeded in 96-well plates in complete culture medium. Treatments were carried out with the four Zn(II) complexes and the five ligands (0.01; 0.05; 0.25; 1.25; 6.25; 31.25; and 156.25 μM) for 48 h. All assays were performed in triplicate. Wells containing only cells and medium (untreated cells) and wells containing cells treated with DMSO diluent were used as experimental controls (considered as 100% cell viability). After incubation, MTT (5 mg/mL-diluted in phosphate-buffered saline—PBS) was added along with complete culture medium at 10% *v*/*v* and maintained for 4 h. Then, 200 μL of DMSO was added to each well and the reading was carried out at 560 nm using the Multiskan FC Microplate Photometer reader (Thermo Fisher Scientific^®^, Waltham, MA, USA).

### 2.7. Clonogenic Assay

Subsequent assays were carried out with complex **4** on the MDA-MB-231 cell line, representative of TNBC, which is known to be more heterogeneous and associated with a worse prognosis [30]. For the experiment, MDA-MB231 cells were seeded in 12-well plates (5.0 × 10^3^ cells/well) and treated with 0.5 or 8.0 µM of complex **4** for 48 h. Afterwards, the treatments were removed, and fresh complete medium was added. The cells were then cultured for 15 days, with the medium changed every three days. For cell fixation, formaldehyde (4% *v*/*v*) and methanol were used. The cells were stained with a crystal violet solution (0.5% *m*/*v*) in methanol (25% *v*/*v*). Colonies were photographed using the iBright Imaging Systems equipment (Thermo Fisher Scientific^®^). Finally, 100 µL of the solution was collected after adding acetic acid (33% *v*/*v*) and transferred to a 96-well plate for absorbance reading at 560 nm on a Multiskan TM FC Microplate Photometer (Thermo Fisher Scientific^®^).

### 2.8. Scratch Wound Healing Assay

To evaluate the effect of complex **4** on cell migration, MDA-MB-231 TNBC cells were seeded in 12-well plates at a density of 2.0 × 10⁵ cells per well. Once the cells reached confluence, a sterile 200 μL pipette tip was used vertically to create a scratch in the cell monolayer, and the cells were treated with 0.5 µM and 8.0 µM of complex **4**. Untreated cells served as controls. Images were captured at 0, 24, 48, and 72 h using the EVOS XL Core Imaging System (Invitrogen^®^, Waltham, MA, USA). The results were analyzed using ImageJ software v. 1.54 g to measure scratch closure, and the percentage of open wound area was plotted over time.

### 2.9. Enzymatic Activity of Caspases 3 and 8

The pro-apoptotic effect of complex **4** was evaluated using a colorimetric assay for the activity of caspases (CAS) 3 and 8. MDA-MB-231 cells were treated with 0.5 µM and 8.0 µM of complex **4** for 48 h. Protein extraction was performed using the NE-PERTM kit (Thermo Fisher Scientific), following the manufacturer’s instructions. Selective substrates for CAS3 (N-acetyl-Asp-Glu-Val-Asp p-nitroanilide, Sigma-Aldrich^®^) and CAS8 (N-acetyl-Ile-Glu-Thr-Asp p-nitroanilide, Sigma-Aldrich^®^) were added at 2 nM to 100 μg of protein in a final volume of 100 μL. After incubation for 16 h, absorbance was measured at 504 nm with a Multiskan TM FC Microplate Photometer (Thermo Fisher Scientific^®^).

### 2.10. qPCR Assay

MDA-MB-231 cells were treated with 0.5 µM of complex **4** for 48 h and total RNA was extracted using Trizol reagent (Invitrogen^®^), following the manufacturer’s protocol. Samples were analyzed for integrity on a 1.5% agarose gel and quantified and qualified using a Nanodrop 1000 spectrophotometer (Thermo Fisher Scientific^®^). For reverse transcription, the M-MLV Reverse Transcriptase kit (Invitrogen^®^) was used and the procedures were carried out according to the supplier’s recommendation. The qPCR assays were conducted using the StepOnePlus system (Applied Biosystems^®^, Waltham, MA, USA) with reactions prepared using a Power SYBR Green PCR Master Mix (Applied Biosystems^®^). Melting curves were monitored and quantification was based on normalization with the reference gene Beta-2-microglobulin (*β2M*). The comparative Cq method was applied to analyze the relative expression of the genes E-Cadherin (*CDH1*), Vimentin (*VIM*), Cytokeratin 18 (*CK18*), Annexin 1 (*ANXA1*), Interleukin 6 (*IL6*), Transforming Growth Factor beta 1 (*TGFβ1*), and Interleukin 1 beta (*IL1β*). The sequences of the primers used are described in Table 1.

### 2.11. Chemosensitization Assay

The ability of complex **4** to sensitize TNBC cells to treatment with Doxorubicin and Paclitaxel was evaluated. For these assays, MDA-MB-231 cells were plated in t-25 tissue culture flasks to reach 80–85% confluence. The cells were pre-treated with complex **4** at a concentration of 0.5 µM for 48 h. Afterwards, the treatments were removed, and the cells were counted and re-plated in 96-well or 24-well plates at densities of 1.5 × 10^4^ and 5.0 × 10^2^ cells per well, respectively. Following adhesion, cells were treated with Doxorubicin (at 2.8, 5.6, and 11.2 µM) or Paclitaxel (at 0.45, 0.9, and 1.81 µM) for 48 h. The effects of these treatments on cell viability or colony formation were determined by using MTT and clonogenic assays as described above. The concentrations of Doxorubicin and Paclitaxel used were based on IC_50_ values that had been previously determined [32].

### 2.12. Statistical Analysis

The data obtained were analyzed using GraphPad Prism 8.0 software. Three independent triplicate assays were conducted and means and standard deviations were calculated. Normality was assessed using the Shapiro–Wilk test and Student’s *t*-test and one-way ANOVA, followed by Tukey’s post-test, were applied to compare treatments. Differences were considered significant when *p* < 0.05. The IC_50_ values were calculated by non-linear regression and the selectivity index (SI) for MDA-MB-231 cells was established by the ratio between the IC_50_ of each non-tumor cell line and the IC_50_ of MDA-MB-231. SI values ≥ 2 were considered significant [33].

## 3. Results and Discussion

### 3.1. Synthesis and Characterization of Zn(II) Complexes

**Scheme 1:**
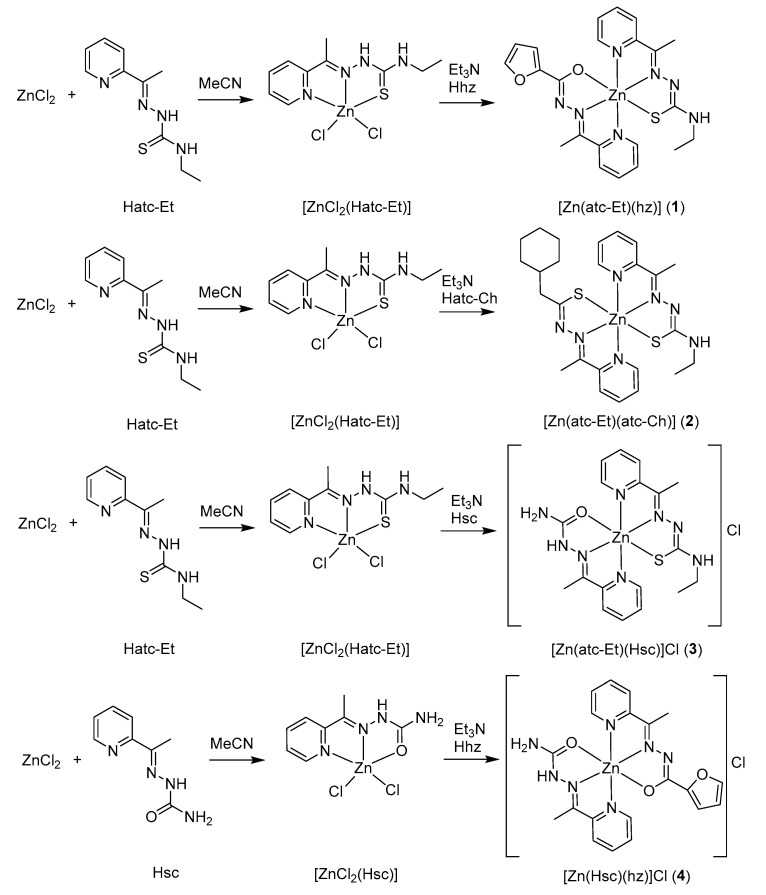
Synthesis of the heteroleptic Zn(II) complexes. Hatc-Ch: 2-Acetylpyridine-4-cyclohexyl-3-thiosemicarbazone; Hatc-Et: 2-Acetylpyridine-4-ethyl-3-thiosemicarbazone; Hsc: 2-Acetylpyridine-semicarbazone; Hhz: 2-Acetylpyridine-furanoylhidrazone.

In this work, four mixed Zn(II) complexes derived from Schiff bases were prepared and fully characterized. The complexes presented a yellow color, were stable to air, and were soluble in dichloromethane, DMSO, and dimethylformamide (DMF) solvents. The complexes were prepared from pentacoordinated complexes, generating [ZnCl_2_(L)] complexes. The chloride ligands in the precursor [ZnCl_2_(L)] complexes were satisfactorily labile to allow ligand substitution reactions. Therefore, it was possible to react one equivalent of the precursors with the same amount of a second ligand in the presence of Et_3_N under reflux in MeCN. Those reactions provided pure microcrystalline precipitates of Zn(II) complexes containing different ligands of the type [Zn(hz)(atc–Et)] (**1**), [Zn(atc–Et)(atc–Ch)] (**2**), [Zn(atc–Et)(Hsc)]Cl (**3**), and [Zn(hz)(Hsc)]Cl (**4**) (see Scheme 1). Two of the new zinc products, complexes **3** and **4**, were cationic. This fact was observed when the reaction was performed, starting from precursors [ZnCl_2_(Hsc)] or [ZnCl_2_(Hatc-Et)] to obtain complex **3** or starting from compounds [ZnCl_2_(Hsc)] or [ZnCl_2_(Hhz)] to acquire complex **4**. In those two cases, even with the addition of Et_3_N, the Hsc ligand remained protonated, which was observed in the IR spectroscopy and after was confirmed by X-ray measurement. Complexes **1** and **2** were formed with the deprotonation of both Htsc-Et and Hhz ligands providing neutral complexes. The cationic products **3** and **4** were pretty soluble in MeOH and complexes **1** and **2** were soluble only in DMSO.

^1^H NMR spectra of **1**–**4** were consistent with the formation of complexes. All spectra presented a set of multiplet signals with similar chemical shifts, in the 6.6–8.5 ppm range, corresponding to aromatic signals from pyridine (for all complexes) and furanoyl (for **1** and **4**) rings. In all spectra, these signals overlapped with those of other aromatic hydrogens, due to similar chemical environments. Integration curves were consistent with the number of hydrogens present in complexes **1**–**4**. Signals at δ 3.6 ppm confirmed the presence of the methyl group in all complexes. Signals at δ 1.14 ppm confirmed the presence of the ethyl group in complexes **1** and **3**, and signals at δ 1.1–1.9 ppm confirmed the presence of ethyl and cyclohexyl groups in complex **2**. In general, the COSY contour map of all complexes showed correlations between the aromatic hydrogens around δ 7.25–8.08 ppm. Besides that, the COSY of complexes **1**–**3** showed the correlations between hydrogen signals δ 1.14 ppm with 3.44 ppm of the ethyl group, while complexes **1** and **4** showed correlations between hydrogen signals δ 6.56 ppm with δ 7.01 and 6.56 ppm with δ 7.75 ppm of the furanoyl group. In additional, the COSY of complex **2** showed the correlations between hydrogen signals δ 1.12 ppm with δ 1.59 and 1.27 ppm with δ 1.72 and 1.25 ppm with δ 1.97 ppm of the cyclohexyl group. Full ^1^H NMR, COSY spectra and signal attribution are provided in the SI section (Supplementary Figures S1–S8).

**Figure 1:**
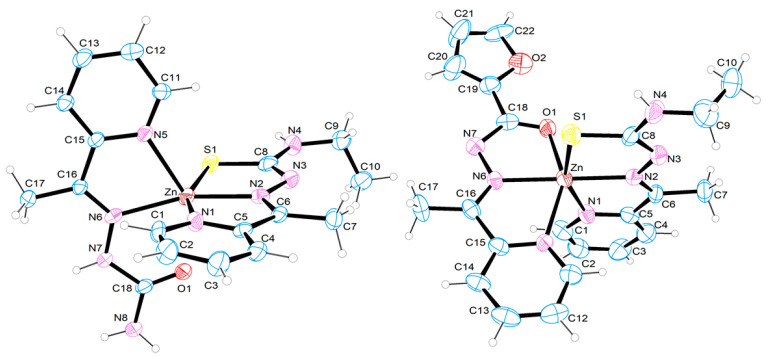
ORTEP view of compounds **1** (**left**) and **3** (**right**) with an atom-numbering scheme and displacement ellipsoids drawn with a 25% probability level and numbering for the X-ray data analyses. The chloride and solvate molecules were omitted for clarity.

X-ray structure analyses confirmed the spectroscopic data. An ORTEP representation of the molecular structure of complex **3** is shown in Figure 1. Selected bond lengths and angles of complex **3** are given in Table S1. Several attempts have been made to obtain crystals of complex **1**, which were not successful to support a discussion about bond lengths and angles. Additonally, it was possible to verify the formation of the mixed ligand complex containing both the thiosemicarbazone and the hydrazone ligands. The coordination environments around the Zn(II) center were best described as distorted octahedral, where the pyridine nitrogen atom N1 and the sulfur S1 occupied the axial positions and the basal plane are defined by the tridentate semicarbazone or hydrazone donor atoms and the azomethine nitrogen N2 of the thiosemicarbazone ligand. This becomes evident when noting the angles N1–Zn–N6 = 93.5° and N1–Zn–S1 = 155.0°. The bond lengths were close to the expected mean values and similar to values found in reported structures [17,34,35]. Interestingly, both the thiosemicarbazone and the hydrazone ligands in complex **1** were deprotonated, while the semicarbazone ligand in **3** remained protonated, affording a cationic complex, as indicated by the spectroscopic and conductimetry data. This is also corroborated by the fact that the C8-S1 and C18-O1 bond lengths had single and double bond characters, respectively. The presence of the chloride in the structure of **3** confirms the formation of the cationic species. This fact also leads to the observation of many H-bonds of the N–H^…^Cl type (Supplementary Table S2 and Figure S9). To our knowledge, these are the first complexes containing a thiosemicarbazone together with a hydrazone or a semicarbazone structurally determined.

### 3.2. Cytotoxicity of Zn(II) Complexes to Cell Lines

**Figure 2:**
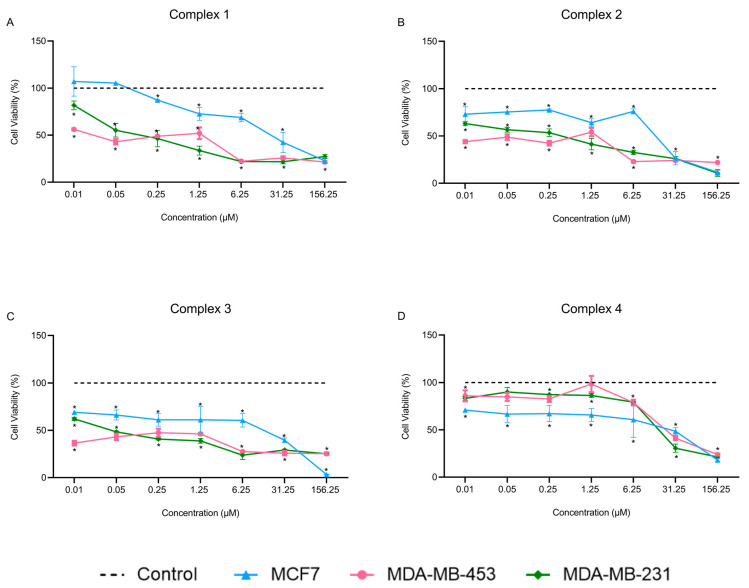
Cytotoxicity of mixed Zn(II) complexes in human breast cancer cell lines MCF7 (ER-positive BC), MDA-MB-453 (HER2-positive BC), and MDA-MB-231 (triple-negative). The treatments were carried out for 48 h. (**A**) Complex **1**; (**B**) Complex **2**; (**C**) Complex **3**; (**D**) Complex **4**. The experiment was performed in triplicate and the results are expressed as mean ± standard deviation. Significance was calculated using ANOVA and Dunnet’s post hoc test. * Indicate statistically significant differences (*p* < 0.05) between treatment and control.

Over the years, different classes of coordinated zinc complexes have been shown potential antitumor effects [36]. Herein, the cytotoxicity of the four new Zn(II) complexes and the five ligands was evaluated. In Figure 2, the percentage of cell viability of the three tumor lineages is shown after 48 h of treatment with complexes **1**–**4**. The ER-positive BC cell line MCF7 proliferated after treatment with the lowest concentrations of complex **1** (Figure 2A) and cell viability decreased when the compound was added at 0.25 µM or in higher concentrations. Complex **1** was cytotoxic to MDA-MB-453 and MDA-MB-231 lineages, especially to the TNBC cells. The cells’ responses to complex **2** (Figure 2B) and complex **3** (Figure 2C) differed from complex **1** mainly for MCF7 cells, with them being active to the ER-positive lineage at the first concentration tested. Finally, for complex **4** (Figure 2D), a slight cytotoxicity to the MDA-MB-231 cells was observed from 0.05 µM, with this being more active at the higher concentrations evaluated.

The cytotoxicity of the ligands individually was also analyzed to confirm the role of mixed complexes for the observed effects (Supplementary Figure S10). For Hatc-Et (Supplementary Figure S10a), the most sensitive lineages were MDA-MB-453 and MDA-MB-231, with MCF7 being the most resistant. For this ligand, the dose-dependent profile in MDA-MB-231 was evident. The Hatc-Ch ligand (Supplementary Figure S10b) proved to be more cytotoxic to the MDA-MB-231 cells when compared to the other lineages. Hhz (Supplementary Figure S10c) decreased the viability of MCF7 and MDA-MB-453 cells only from 6.25 µM, with it being cytotoxic to MDA-MB-231 already at a concentration of 1.25 µM. For Hsc (Supplementary Figure S10d) and ZnCl_2_ (Supplementary Figure S10e), only MDA-MB-453 cells were sensitive.

The half-maximal inhibitory concentration (IC_50_) values for the mixed Zn(II) complexes and their ligands are shown in Table 2. For the representative TNBC MDA-MB231, the ligands of complex **4** were less active. In this case, cytotoxicity is related to the complex and not to its ligands individually.

**Figure 3:**
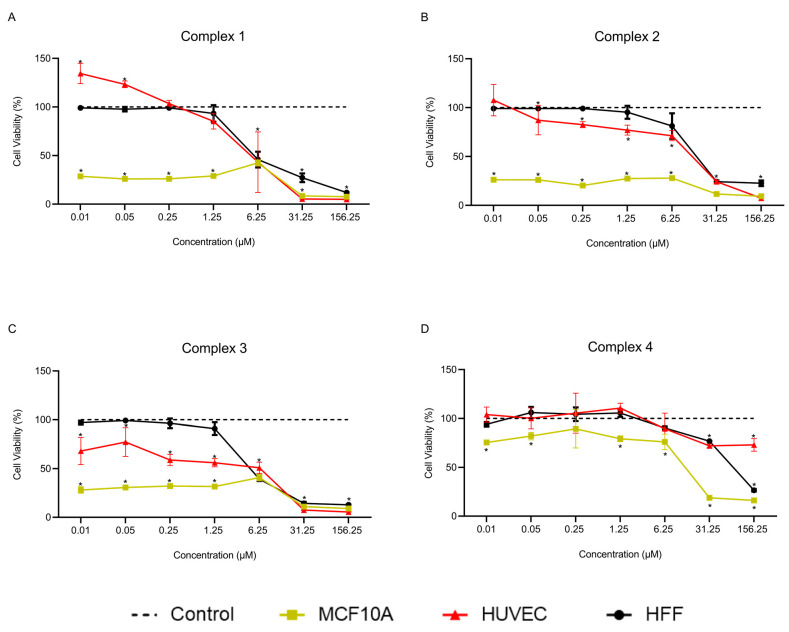
Cytotoxicity of mixed Zn(II) complexes on non-tumor human cell lines MCF10A (mammary epithelial cell line), HUVEC (umbilical vein endothelial cells), and HFF (fibroblasts). Treatments were performed for 48 h. (**A**) Complex **1;** (**B**) Complex **2**; (**C**) Complex **3**; (**D**) Complex **4**. The experiment was performed in triplicate and the results are expressed as mean ± standard deviation. Significance was calculated using ANOVA and Dunnet’s post hoc test. * Indicate statistically significant differences (*p* < 0.05) between treatment and control.

Subsequently, the cytotoxicity of complexes **1**–**4** was evaluated in the non-tumor cell lines MCF10A, HUVEC, and HFF (Figure 3). MCF10A cells were the most sensitive, especially when treated with complex **1**, complex **2,** and complex **3**. For HUVEC and HFF cells, a significant decrease in viability was observed for 6.25 µM of complex **1**, (Figure 3A), complex **2** (Figure 3B), and complex **3** (Figure 3C). However, for 6.25 µM of complex **4**, the viability of non-tumor cells remained above 50%.

Complexes **1**–**3** showed IC_50_ values for MCF-10A cells lower than 0.01 µM (the lowest concentration tested, Table 3), and for HUVEC cells, complex **4** was the most selective. Therefore, complex **4** was selected for subsequent assays on the MDA-MB231 cell line, since it was active, selective, and its ligands individually did not alter cell viability.

### 3.3. Colony Formation and Migration of MDA-MB-231 Cells

As MDA-MB-231 cells are representative of aggressive breast tumors, the effects of complex **4** on their clonogenicity were evaluated. Clonogenicity refers to the potential of tumor cells to migrate to other tissues and initiate new colonies [37]. The concentrations used in these experiments were defined based on values proportional to the IC_50_, specifically, IC_50_/2 (8.0 µM) and IC_50_/32 (0.5 µM). The lowest concentration was chosen to ensure that non-tumor cell viability remained around 100% at 0.5 µM.

**Figure 4:**
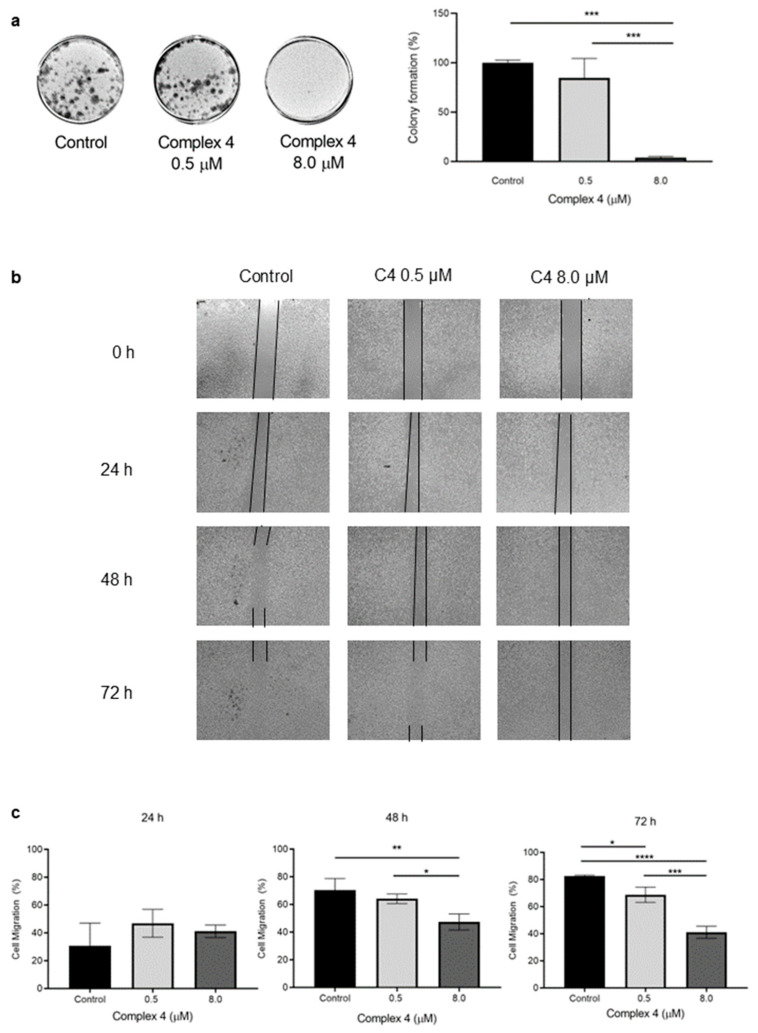
Effect of complex **4** on the clonogenicity and migration of the MDA-MB-231 cell line. Cells were treated with 0.5 µM and 8.0 µM of complex **4**. (**a**) Representative images of colonies formed in the wells and the quantification of the colony formation of MDA-MB-231 cells after 48 h of treatment. Colonies were photographed using iBright Imaging Systems (Thermo Fisher Scientific®) equip-ment that provides life-size images. (**b**) Representative images of cell migration. Magnification: 40×. (**c**) Represents the graphs of the quantification of wound closure after 24, 48 and 72 h of treatment. The experiments were performed in triplicate and the results are expressed as mean ± standard deviation. Significance was calculated using ANOVA and Tukey’s post hoc test (* *p* < 0.05; ** *p* < 0.01; *** *p* < 0.0001; **** *p* < 0.00001).

The results, shown in Figure 4a, indicated a slight inhibition in colony formation at the lowest concentration. However, treatment with complex **4** at 8.0 µM resulted in a significant reduction in the number of colonies.

Next, the results of the wound healing assay (Figure 4b,c) were consistent with those of the clonogenicity assay, demonstrating that complex **4** significantly inhibited the migration of MDA-MB-231 cells. Interestingly, after 72 h of treatment, significant inhibition of cell migration was observed even at the lowest concentration of complex **4** (0.5 µM), indicating a time-dependent effect.

Thus, complex **4** significantly inhibited both the clonogenicity and migration of MDA-MB-231 cells, which are crucial events in tumor progression and metastasis [37].

### 3.4. Enzymatic Activity of CAS3 and CAS8

**Figure 5:**
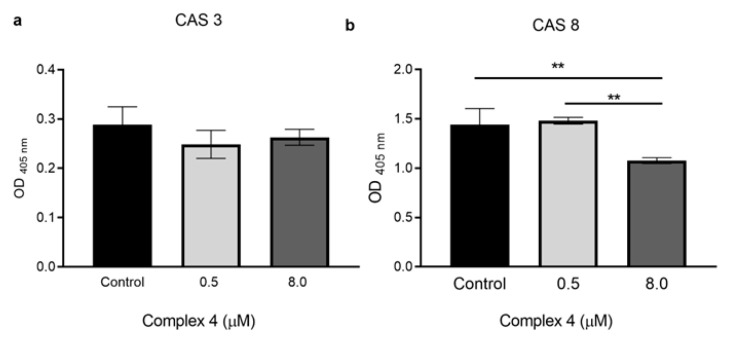
Assessment of the activity of caspase 3 (CAS 3) and caspase 8 (CAS8) in the MDA-MB-231 cell line. Cells were treated with 0.5 µM and 8.0 µM of complex **4**. Quantification was performed by measuring optical density (OD) at 405 nm. The results are expressed as mean ± standard deviation. Significance was calculated using ANOVA and Tukey’s post hoc test (** *p* < 0.01). (**a**) Quantification of CAS3 activity. (**b**) Quantification of CAS 8 activity.

The activities of the caspase enzymes CAS3 and CAS8 were also evaluated following treatments of MDA-MB-231 cells with complex **4**. No change was observed in the activity of CAS3 (Figure 5a). However, the activity of CAS8 (Figure 5b) decreased when the cells were treated with 8.0 µM of the compound. Our results indicate that complex **4** can induce necroptosis in TNBC cells, a death mechanism that overcomes apoptosis resistance and triggers antitumor immunity [38].

### 3.5. Transcriptional Modulation Mediated by Treatment with Complex ***4***

**Figure 6:**
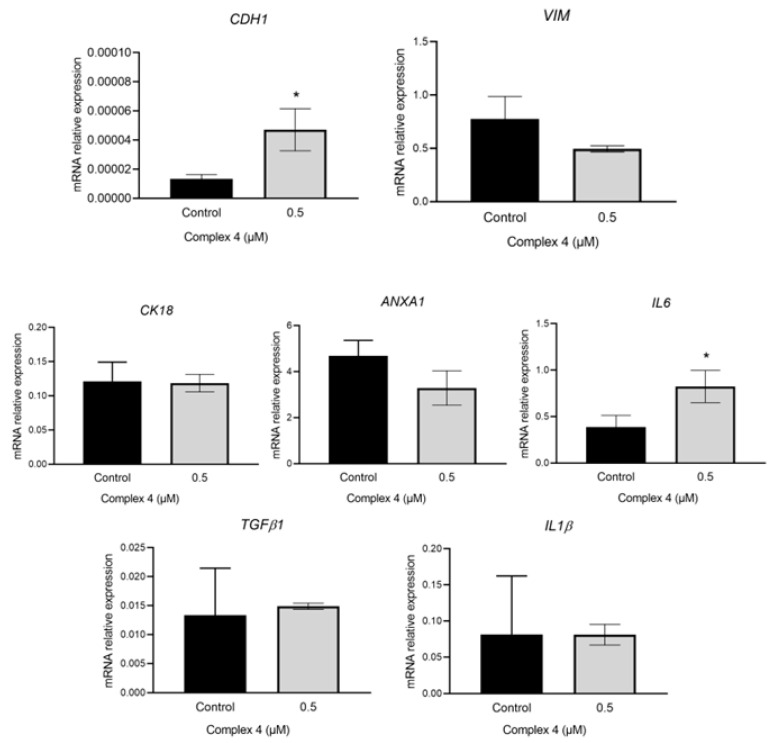
Relative expression of transcriptional levels of E-Cadherin (*CDH1*), Vimentin (*VIM*), Cytokeratin 18 (*CK18*), Annexin A1 (*ANXA1*), Interleukin 6 (*IL6*), Transforming Growth Factor beta 1 (*TGFβ1*), and Interleukin 1 beta (*IL1β*) after treating MDA-MB-231 cells with 0.5 µM of complex **4** for 48 h. The results are expressed as mean ± standard deviation. Significance was calculated using Student’s *t*-test (* *p* < 0.05).

Transcriptional levels of *CDH1*, *VIM*, *CK18, ANXA1*, *IL6*, *TGFβ1,* and *IL1β* were quantified to evaluate the modulatory effects of complex **4** on MDA-MB-231 cell transcripts (Figure 6). Treatment with 0.5 µM of the compound increased the gene expression of *CDH1* and *IL6*. For the other targets, no statistically significant differences were observed, although there was a trend toward decreased *VIM* and *ANXA1* transcripts. The increased *CDH1* expression suggests that complex **4** can modulate epithelial–mesenchymal transition (EMT). Additionally, the increased *IL6* expression may be a response to the decrease in *ANXA1* transcripts, as the cells attempt to restore AnxA1 autocrine signaling in TNBC [7,39].

### 3.6. Complex ***4*** Sensitizes MDA-MB231 Cells to Treatment with Doxorubicin and Paclitaxel

**Figure 7:**
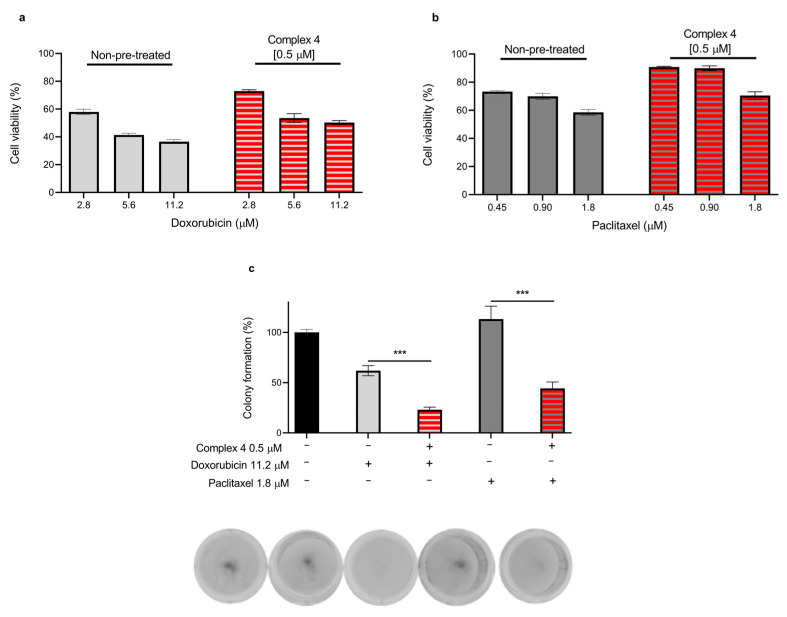
Chemosensitization assay performed on MDA-MB-231 cells pretreated with 0.5 µM of complex **4**. Results are expressed as mean ± standard deviation. (**a**) Viability of cells pretreated with complex **4** and subsequently treated with Doxorubicin. (**b**) Viability of cells pretreated with complex **4** and subsequently treated with Paclitaxel. (**c**) Assessment of colony formation of cells pretreated with complex **4** and subsequently treated with Doxorubicin and Paclitaxel. Colonies were photographed using iBright Imaging Systems (Thermo Fisher Scientific®) equipment that provides life-size images. Significance was calculated using ANOVA and Student’s *t* test (*** *p* < 0.0001).

Multidrug resistance is one of the main causes of chemotherapy failure [40]. Therefore, we evaluated whether complex **4** sensitizes MDA-MB-231 cells to the chemotherapy drugs Doxorubicin and Paclitaxel (Figure 7). Pretreatment with 0.5 µM of complex **4** for 48 h did not significantly alter cell viability (Figure 7a,b). However, when these cells were pretreated with complex **4**, the clonogenicity of the TNBC lineage was significantly reduced by both chemotherapeutics. This inhibition was more evident for Doxorubicin (Figure 7c).

Despite the complexity of the events related to therapeutic failure, our results expand the understanding of the mechanisms underlying the response of TNBC cells to Zn complexes, suggesting that complex **4** may be particularly promising. Recently, Rudolf and colleagues [41] showed that the Zn intracellular content in hiPCS-CM cells has pleiotropic effects on Doxorubicin toxicity. Furthermore, Xue et al. [42] demonstrated that Zn promotes prostate cancer cell chemosensitivity to Paclitaxel through the modulation of EMT markers, which reinforces the data from our study.

## 4. Conclusions

Chemotherapy remains the primary strategy for treating TNBC. However, these tumors tend to progress rapidly, as conventional chemotherapies are often insufficient to suppress tumor-associated chemoresistance. New compounds are needed, and medicinal chemistry has emerged as a field proposing optimized structures. The present work reports a new Zn(II) complex with a Schiff base, complex **4**, as a promising agent for TNBC management. This compound was the most active and selective for TNBC cells, in addition to inhibiting colony formation and migration. Importantly, complex **4** sensitized TNBC cells to Doxorubicin and Paclitaxel, upregulating CDH1 transcripts. According to the results presented here, this signaling pathway may be modulated by Zn(II) complexes with Schiff bases, affecting the EMT mechanism and, consequently, the TNBC resistance phenotype.

## Figures and Tables

**Table 1:** Sequence of primers used for each target, including E-Cadherin (CDH1), Vimentin (VIM), Cytokeratin 18 (CK18), Annexin 1 (ANXA1), Interleukin 6 (IL6), Transforming Growth Factor beta 1 (TGFβ1), and Interleukin 1 beta (IL1β) [31].

Gene	Sequence (Forward–Reverse) 5′–3′	Amplicon (pb)
*CDH1*	F:CTGGCGTCTGTAGGAAGGC R:GCTGGCTCAAGTCAAAGTCCTG	240
*VIM*	F:GAGACGCATTGTCAACATCCTG R:CAAGAACACCCGCACCAAC	356
*CK18*	F:GCTCTGGGTTGACCGTGG R:GTGGTGCTCTCCTCAATCTGC	151
*ANXA1*	F:GATTCAGATGCCAGGGCCT R:CACTCTGCGAAGTTGTGGAT	111
*IL6*	F:GATTCCAAAGATGTAGCCGCC R:ATTTTCACCAGGCAAGTCTCCTC	
*TGFβ1*	F:GTACCTGAACCCGTGTTGCTC R:CAGGAATTGTTGCTGTATTTCTGG	107
*IL1β*	F:ACAGGATATGGAGCAACAAGTGG R:GGGCTTATCATCTTTCAACACGC	136
*β2M*	F:CCTGCCGTGTGAAC-CATGT R:CGGCATCTTCAAACCTCC	94

**Table 2:** Values of the concentration that inhibits 50% of cell viability (IC_50_) after treatment of mammary tumor cell lines with mixed Zn(II) complexes and their ligands for 48 h.

	IC_50_ (μM)		
	MCF7	MDA-MB-453	MDA-MB-231
Complex 1	9.43	0.32	0.24
Complex 2	18.49	<0.01	0.92
Complex 3	19.34	<0.01	0.16
Complex 4	10.41	12.82	16.3
Hatc-Et	NA	<0.01	0.27
Hatc-Ch	NA	NA	6.25
Hhz	NA	4.11	30.3
Hsc	NA	NA	NA
ZnCl_2_	NA	<0.01	NA

NA: IC_50_ > 156.25 µM.

**Table 3:** Concentration values that inhibit 50% of cell viability (IC_50_) for the treatment of non-tumor cell lines with mixed Zn(II) complexes for 48 h. The selectivity index (SI) is also presented. The SI was calculated by dividing the IC_50_ value of each complex in non-tumor cells by the IC_50_ of MDA-MB-231 cells.

	IC_50_ (μM)			SI (Non-Tumor/MDA-MB231)		
	MCF10A	HUVEC	HFF	MCF10A	HUVEC	HFF
Complex 1	<0.01	2.52	4.57	NS	10.50	19.04
Complex 2	<0.01	20.38	9.49	NS	22.15	10.31
Complex 3	<0.01	10.09	4.22	NS	63.06	26.37
Complex 4	12.54	NA	131.25	0.77	HS	8.05

NA: IC_50_ > 156.25 µM; NS: nonselective; HS: highly selective.

## Data Availability

The original contributions presented in this study are included in the article/Supplementary Material. Further inquiries can be directed to the corresponding authors.

## Supplementary Materials

The following supporting information can be downloaded at: https://www.mdpi.com/article/10.3390/pharmaceutics16121610/s1: Figure S1: ^1^H NMR spectrum (400 MHz, DMSO) of complex **1**; Figure S2: COSY contour map (δ, DMSO, 400 MHz) of complex **1**; Figure S3: ^1^H NMR spectrum (400 MHz, DMSO) of complex **2**; Figure S4: COSY contour map (δ, DMSO, 400 MHz) of complex **2**; Figure S5: ^1^H NMR spectrum (400 MHz, DMSO) of complex **3**; Figure S6: COSY contour map (δ, DMSO, 400 MHz) of complex **3**; Figure S7: ^1^H NMR spectrum (400 MHz, DMSO) of complex **4**; Figure S8: COSY contour map (δ, DMSO, 400 MHz) of complex **4**; Figure S9: Structural packing of complex **3** in the b axis direction. Dashed lines blue and green indicates C–H…O hydrogen bond and C–H…π interaction, respectively; Figure S10: Cytotoxicity of ligands of mixed Zn(II) complexes in MCF7 (ER-positive breast cancer), MDA-MB-453 (HER2-positive breast cancer), and MDA-MB-231 (triple-negative breast cancer) cell lines. The treatment was carried out for 48 h. (**a**) Hatc–Et. (**b**) Hhz. (**c**) Hhsc. (**d**) Hatc-Ch. (**e**) ZnCl_2_. The experiments were performed in triplicate and the results are expressed as mean ± standard deviation. Significance was calculated by ANOVA and Tukey’s post hoc test. Letters represent significance (*p* < 0.05) between cell lines. (**a**) Control × MCF7, (**b**) Control × MDA-MB-453, (**c**) Control × MDA-MB-231, (**d**) MCF7 × MDA-MB-453, (**e**) MCF7 × MDA-MB-231, and (**f**) MDA-MB-453 × MDA-MB-231; Table S1: Selected bond lengths (Å) and angles (°) for complex **3**; Table S2: Hydrogen–bond geometry (Å, °) in the crystal structure of **3**. Table S3: Crystallographic data for **3**.

## References

[1] WHO Breast Cancer. https://www.who.int/news-room/fact-sheets/detail/breast-cancer.

[2] Alagoz O., Lowry K.P. (2021). Impact of the COVID-19 Pandemic on Breast Cancer Mortality in the US: Estimates From Collaborative Simulation Modeling. JNCI J. Natl. Cancer Inst..

[3] Harbeck N., Penault-Llorca F., Cortes J., Gnant M., Houssami N., Poortmans P., Ruddy K., Tsang J., Cardoso F. (2019). Breast cancer. Nat. Rev. Dis. Primers.

[4] Bai X., Ni J., Beretov J., Graham P., Li Y. (2021). Triple-negative breast cancer therapeutic resistance: Where is the Achilles’ heel?. Cancer Lett..

[5] Yin L., Duan J.J., Bian X.W., Yu S.C. (2020). Triple-negative breast cancer molecular subtyping and treatment progress. Breast Cancer Res..

[6] McGee S.F. (2023). Understanding Metastasis: Current Paradigms and Therapeutic Challenges in Breast Cancer Progression.

[7] Qu Z., Liu Q., Kong X., Wang X., Wang Z., Wang J., Fang Y. (2023). A Systematic Study on Zinc-Related Metabolism in Breast Cancer. Nutrients.

[8] Rusch P., Hirner A.V., Schmitz O., Kimmig R., Hoffmann O., Diel M. (2021). Zinc distribution within breast cancer tissue of different intrinsic subtypes. Arch. Gynecol. Obstet..

[9] Chandler P., Kochupurakkal B.S., Alam S., Richardson A.L., Soybel D.I., Kelleher S.L. (2016). Subtype-specific accumulation of intracellular zinc pools is associated with the malignant phenotype in breast cancer. Mol. Cancer.

[10] Geraki K., Farquharson M.J., Bradley D.A. (2002). Concentrations of Fe, Cu and Zn in breast tissue: A synchrotron XRF study. Phys. Med. Biol..

[11] Fahmy H.M., Mosleh A.M., El-Sayed A.A., El-Sherif A.A. (2023). Novel palladium(II) and Zinc(II) Schiff base complexes: Synthesis, biophysical studies, and anticancer activity investigation. J. Trace Elem. Med. Biol. Organ Soc. Miner. Trace Elem. (GMS).

[12] Manikandamathavan V.M., Weyhermüller T., Parameswari R.P., Sathishkumar M., Subramanian V., Nair B.U. (2014). DNA/protein interaction and cytotoxic activity of imidazole terpyridine derived Cu(II)/Zn(II) metal complexes. Dalton Trans..

[13] Narwane M., Dorairaj D.P., Chang Y.L. (2021). Tris-(2-pyridyl)-pyrazolyl Borate Zinc(II) Complexes: Synthesis, DNA/Protein Binding and In Vitro Cytotoxicity Studies. Molecules.

[14] Mahadevi P., Sumathi S. (2023). Schiff base metal complexes: Synthesis, optoelectronic, biological studies, fabrication of zinc oxide nanoparticles and its photocatalytic activity. Results Chem..

[15] Bashir M., Dar A.A., Yousuf I. (2023). Syntheses, Structural Characterization, and Cytotoxicity Assessment of Novel Mn(II) and Zn(II) Complexes of Aroyl-Hydrazone Schiff Base Ligand. ACS Omega.

[16] Çakmak R., Ay B., Çınar E., Başaran E., Akkoç S., Boğa M., Taş E. (2023). Synthesis, spectroscopic, thermal analysis and in vitro cytotoxicity, anticholinesterase and antioxidant activities of new Co (II), Ni (II), Cu (II), Zn (II), and Ru (III) complexes of pyrazolone-based Schiff base ligand. J. Mol. Struct..

[17] Lopes E.D.O., Oliveira C.G.d., Silva P.B.d., Eismann C.E., Suárez C.A., Menegário A.A., Leite C.Q.F., Deflon V.M., Pavan F.R. (2016). Novel Zinc(II) Complexes [Zn(atc-Et)2] and [Zn(atc-Ph)2]: In Vitro and in Vivo Antiproliferative Studies. Int. J. Mol. Sci..

[18] Lobana T.S., Kumari P., Hundal G., Butcher R.J., Castineiras A., Akitsu T. (2013). Metal derivatives of N1-substituted thiosemicarbazones: Synthesis, structures and spectroscopy of nickel (II) and cobalt (III) complexes. Inorg. Chim. Acta.

[19] Pavan F.R., Maia P.I.d.S., Leite S.R., Deflon V.M., Batista A.A., Sato D.N., Franzblau S.G., Leite C.Q. (2010). Thiosemicarbazones, semicarbazones, dithiocarbazates and hydrazide/hydrazones: Anti–Mycobacterium tuberculosis activity and cytotoxicity. Eur. J. Med. Chem..

[20] Kovala-Demertzi D., Yadav P.N., Wiecek J., Skoulika S., Varadinova T., Demertzis M.A. (2006). Zinc (II) complexes derived from pyridine-2-carbaldehyde thiosemicarbazone and (1E)-1-pyridin-2-ylethan-1-one thiosemicarbazone. Synthesis, crystal structures and antiproliferative activity of zinc (II) complexes. J. Inorg. Biochem..

[21] Bermejo E., Carballo R., Castiñeiras A., Domĺnguez R., Maichle-Mössmer C., Strähle J., West D.X. (1999). Synthesis, characterization and antifungal activity of group 12 metal complexes of 2-acetylpyridine-4N-ethylthiosemicarbazone (H4EL) and 2-acetylpyridine-N-oxide-4N-ethylthiosemicarbazone (H4ELO). Polyhedron.

[22] Kasuga N.C., Sekino K., Ishikawa M., Honda A., Yokoyama M., Nakano S., Shimada N., Koumo C., Nomiya K. (2003). Synthesis, structural characterization and antimicrobial activities of 12 zinc (II) complexes with four thiosemicarbazone and two semicarbazone ligands. J. Inorg. Biochem..

[23] (2001).

[24] Sheldrick G.M. (2008). A short history of SHELX. Acta Crystallogr. Sect. A Found. Crystallogr..

[25] Sheldrick G.M. (2015). SHELXT–Integrated space-group and crystal-structure determination. Acta Crystallogr. Sect. A Found. Adv..

[26] Sheldrick G.M. (2015). Crystal structure refinement with SHELXL. Acta Crystallogr. Sect. C Struct. Chem..

[27] Dolomanov O.V., Bourhis L.J., Gildea R.J., Howard J.A., Puschmann H. (2009). OLEX2: A complete structure solution, refinement and analysis program. J. Appl. Crystallogr..

[28] Farrugia L.J. (2012). WinGX and ORTEP for Windows: An update. J. Appl. Crystallogr..

[29] Macrae C.F., Edgington P.R., McCabe P., Pidcock E., Shields G.P., Taylor R., Towler M., Streek J. (2006). Mercury: Visualization and analysis of crystal structures. J. Appl. Crystallogr..

[30] Huang Z., Yu P., Tang J. (2020). Characterization of triple-negative breast cancer MDA-MB-231 cell spheroid model. OncoTargets Ther..

[31] Araujo T.G., Marangoni K., Rocha R.M., Maia Y.C., Araujo G.R., Alcântar T.M., Alves P.T., Calábria L., Neves A.F., Soares F.A. (2014). Dynamic dialog between cytokeratin 18 and annexin A1 in breast cancer: A transcriptional disequilibrium. Acta Histochem..

[32] Ferreira H.S.V., Ramos L.M.S., Silva F.C., Alves D.L., de Menezes Pereira G., de Oliveira Santiago P.H., de Almeida A.M., Ellena J., Corbi P.P., Oliveira C.G. (2024). A new copper (II) complex containing long-chain aliphatic hydrazide and 1, 10-phenanthroline upregulates ADP hydrolysis in triple-negative breast cancer cells. J. Inorg. Biochem..

[33] Badisa R.B., Darling-Reed S.F., Joseph P., Cooperwood J.S., Latinwo L.M., Goodman C.B. (2009). Selective cytotoxic activities of two novel synthetic drugs on human breast carcinoma MCF-7 cells. Anticancer Res..

[34] Souza R.A.C., Cunha V.L., de Faria Franca E., Deflon V.M., Maia P.I., Oliveira C.G. (2022). Synthesis, Structural Characterization, X-ray, Hirshfeld Surfaces, DFT calculations, In Silico ADME Approach and a Molecular Docking Study of a New Nickel (II) Complex. ChemistrySelect.

[35] Souza R.A., Cunha V.L., de Souza J.H., Martins C.H., Franca E.d.F., Pivatto M., Ellena J.A., Faustino L.A., Patrocinio A.O.d.T., Deflon V.M. (2022). Zinc (II) complexes bearing N, N, S ligands: Synthesis, crystal structure, spectroscopic analysis, molecular docking and biological investigations about its antifungal activity. J. Inorg. Biochem..

[36] Porchia M., Pellei M., Del Bello F., Santini C. (2020). Zinc complexes with nitrogen donor ligands as anticancer agents. Molecules.

[37] Balakrishnan N., Haribabu J., Dhanabalan A.K., Swaminathan S., Sun S., Dibwe D.F., Bhuvanesh N., Awale S., Karvembu R. (2020). Thiosemicarbazone (s)-anchored water soluble mono-and bimetallic Cu (II) complexes: Enzyme-like activities, biomolecular interactions, anticancer property and real-time live cytotoxicity. Dalton Trans..

[38] Gong Y., Fan Z., Luo G., Yang C., Huang Q., Fan K., Cheng H., Jin K., Ni Q., Yu X. (2019). The role of necroptosis in cancer biology and therapy. Mol. Cancer.

[39] Vecchi L., Mota S.T.S., Zóia M.A.P., Martins I.C., de Souza J.B., Santos T.G., Beserra A.d.O., de Andrade V.P., Goulart L.R., Araújo T.G. (2022). Interleukin-6 signaling in triple negative breast cancer cells elicits the annexin A1/formyl peptide receptor 1 axis and affects the tumor microenvironment. Cells.

[40] Abd-Ellatef G.E.F., Gazzano E., El-Desoky A.H., Hamed A.R., Kopecka J., Belisario D.C., Costamagna C., Marie M.A.S., Fahmy S.R., Abdel-Hamid A.-H.Z. (2022). Glabratephrin reverses doxorubicin resistance in triple negative breast cancer by inhibiting P-glycoprotein. Pharmacol. Res..

[41] Rudolf K., Rudolf E. (2023). Increased Intracellular Free Zinc Has Pleiotropic Effects on Doxorubicin-Induced Cytotoxicity in hiPCS-CMs Cells. Int. J. Mol. Sci..

[42] Xue Y.N., Yu B.B., Liu Y.N., Guo R., Li J.L., Zhang L.C., Su J., Sun L.K., Li Y. (2019). Zinc promotes prostate cancer cell chemosensitivity to paclitaxel by inhibiting epithelial-mesenchymal transition and inducing apoptosis. Prostate.

