# Supplementary material for: A New Heteroleptic Zn(II) Complex with Schiff Bases Sensitizes Triple-Negative Breast Cancer Cells to Doxorubicin and Paclitaxel
Source: Pharmaceutics. 2024 Dec 18;16(12):1610. doi: 10.3390/pharmaceutics16121610 (PMC11676115; doi:10.3390/pharmaceutics16121610)
Supplement: Supplementary file 1 [file pharmaceutics-16-01610-s001.zip › pharmaceutics-3350768-supplementary.pdf]

A new heteroleptic Zn(II) complex with Schiff bases sensitizes triple-negative breast cancer cells to Doxorubicin and Paclitaxel

Raiane Aparecida dos Santos Machado[1], Raoni Pais Siqueira[1], Fernanda Cardoso da Silva[1], André Carlos Pereira de Matos[1], Dayanne Silva Borges[1], Gislaine Gonçalves Rocha[1], Thais Cristina Prado de Souza[2], Rafael Aparecido Carvalho Souza[2], Clayton Rodrigues de Oliveira[3], Antônio G. Ferreira[3], Pedro Ivo da Silva Maia[4], Victor Marcelo Deflon[5], Carolina Gonçalves Oliveira[2*] and Thaise Gonçalves Araújo[1,6*]

[1]Laboratory of Genetics and Biotechnology, Institute of Biotechnology, Universidade Federal de Uberlândia, Patos de Minas-MG 38700-002, Brazil; machado.raiane@hotmail.com (R.A.S.M.); raoni.siqueira@ufu.br (R.P.S.); fernanda.cardoso95@yahoo.com (F.C.S.); andre3ptm@gmail.com (A.C.P.M.); daaysborges@gmail.com (D.S.B.); gislaineg.rocha08@gmail.com (G.G.R.); tgaraujo@ufu.br (T.G.A.).

[2]Institute of Chemistry, Universidade Federal de Uberlândia, Uberlândia, MG, 38400-902, Brazil; ; psthaiscristina@gmail.com (T.C.P.S.); rafasouza27@hotmail.com (R.A.C.S.)

[3]Department of Chemistry, Universidade Federal de São Carlos São Carlos, SP, Brazil, Rodovia Washington Luís km 235, 13565-905; claytonrol@hotmail.com (C.R.O); giba@ufscar.br (A.G.F)

[4]Bioactive Compounds Development Research Group, Universidade Federal do Triângulo Mineiro, Av. Dr. Randolfo Borges 1400, Uberaba 38025-440, MG, Brazil; pedro.maia@uftm.edu.br (P.I.S.M.).

[5]São Carlos Institute of Chemistry, Universidade de São Paulo, São Carlos, SP, 13560-970 Brazil; deflon@iqsc.usp.br (V.M.D.)

[6]Laboratory of Nanobiotechnology Prof. Dr. Luiz Ricardo Goulart Filho, Institute of Biotechnology, Universidade Federal de Uberlândia, Uberlandia-MG, 38405-302, Brazil.

Correspondence: C. G. O, carolina@ufu.br. Tel.: +55 34 999979271; Group of Bioinorganic Chemistry, Institute of Chemistry, Federal University of Uberlândia, Uberlândia, MG, Brazil. T.G.A, tgaraujo@ufu.br; Tel.: +55 34 38142027; Laboratory of Genetics and Biotechnology, Institute of Biotechnology, Universidade Federal de Uberlândia, Rua Major Jerônimo, 566, Sala 601, 38700-002, Patos de Minas, MG, Brazil.

CONTENTS

Figure S1: ^{1}H NMR spectrum (400 MHz, DMSO) of Complex **1**. 3
Figure S2: COSY contour map (δ, DMSO, 400 MHz) of Complex **1**. 3
Figure S3: ^{1}H NMR spectrum (400 MHz, DMSO) of Complex **2**. 3
Figure S4: COSY contour map (δ, DMSO, 400 MHz) of Complex **2**. 4
Figure S5: ^{1}H NMR spectrum (400 MHz, DMSO) of Complex **3**. 5
Figure S6: COSY contour map (δ, DMSO, 400 MHz) of Complex **3**. 5
Figure S7: ^{1}H NMR spectrum (400 MHz, DMSO) of Complex **4**. 6
Figure S8: COSY contour map (δ, DMSO, 400 MHz) of Complex **4**. 6
Table S1. Selected bond lengths (Å) and angles (°) for complex **3**. 7
Figure S9: Structural packing of Complex **3** in the *b* axis direction. Dashed lines blue and green indicates C–H···O hydrogen bond and C–H···π interaction, respectively. 7
Table S2. Hydrogen–bond geometry (Å, °) in the crystal structure of **3**. 7
Figure S10: Cytotoxicity of ligands of mixed Zn(II) complexes in MCF7 (ER-positive breast cancer), MDA-MB-453 (HER2-positive breast cancer) and MDA-MB-231 (triple-negative breast cancer) cell lines. The treatment was carried out for 48 hours. (a) Hatc – Et. (B) Hhz. (c) Hhsc. (d) Hatc - Ch. (e) $ZnCl_2$. The experiments were performed in triplicate and the results are expressed as mean ± standard deviation. Significance was calculated by ANOVA and Tukey's post-hoc test. Letters represent significance ($p < 0.05$) between cell lines. a) Control x MCF7, b) Control x MDA-MB-453, c) Control x MDA-MB-231, d) MCF7 x MDA-MB-453, e) MCF7 x MDA-MB-231 and f) MDA-MB -453 x MDA-MB-231. 8
Table S3. Crystallographic data for **3**. 9

Figure S1: ^{1}H NMR spectrum (400 MHz, DMSO) of Complex **1**.

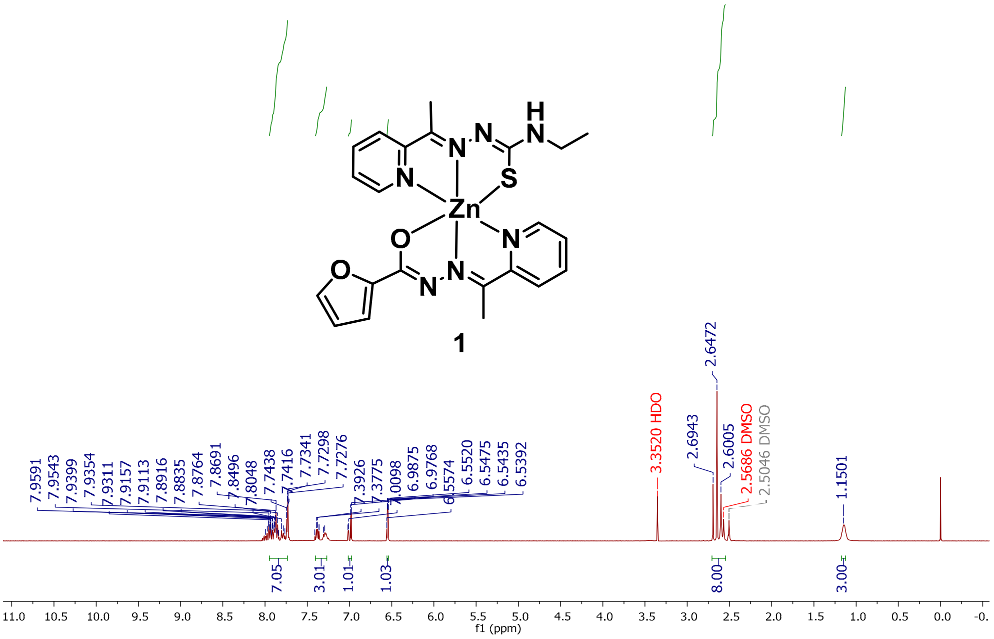

Figure S2: COSY contour map (δ, DMSO, 400 MHz) of Complex **1**.

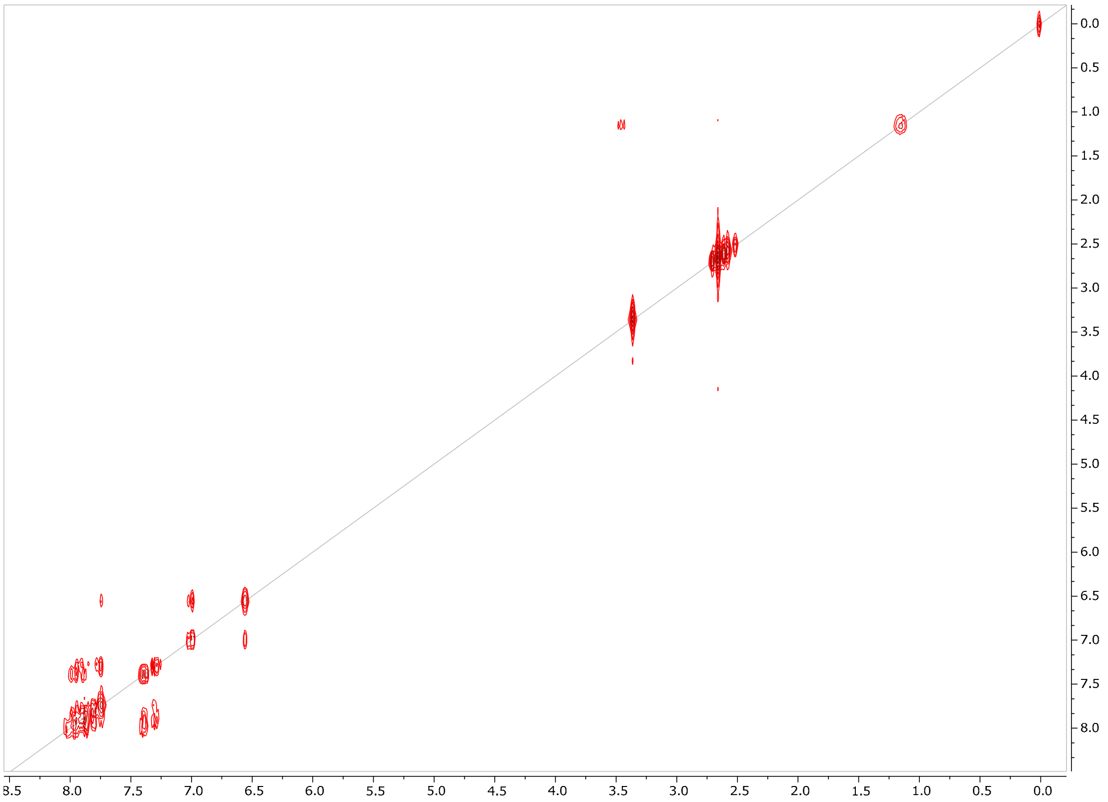

Figure S3: ^{1}H NMR spectrum (400 MHz, DMSO) of Complex **2**.

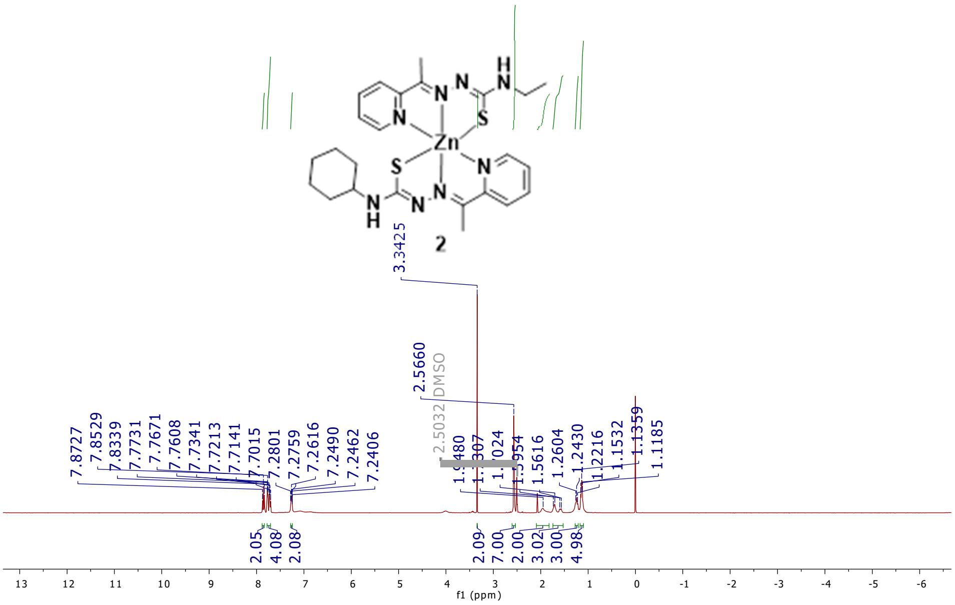

Figure S4: COSY contour map (δ, DMSO, 400 MHz) of Complex **2**.

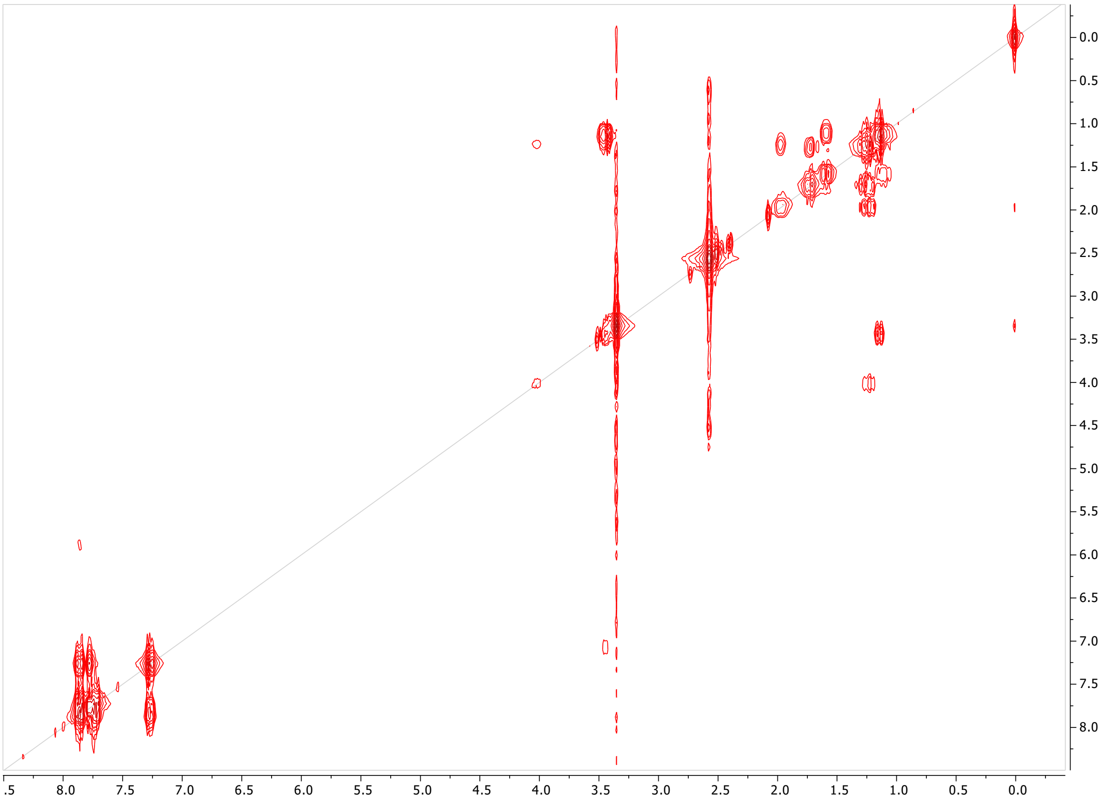

Figure S5: ^{1}H NMR spectrum (400 MHz, DMSO) of Complex **3**.

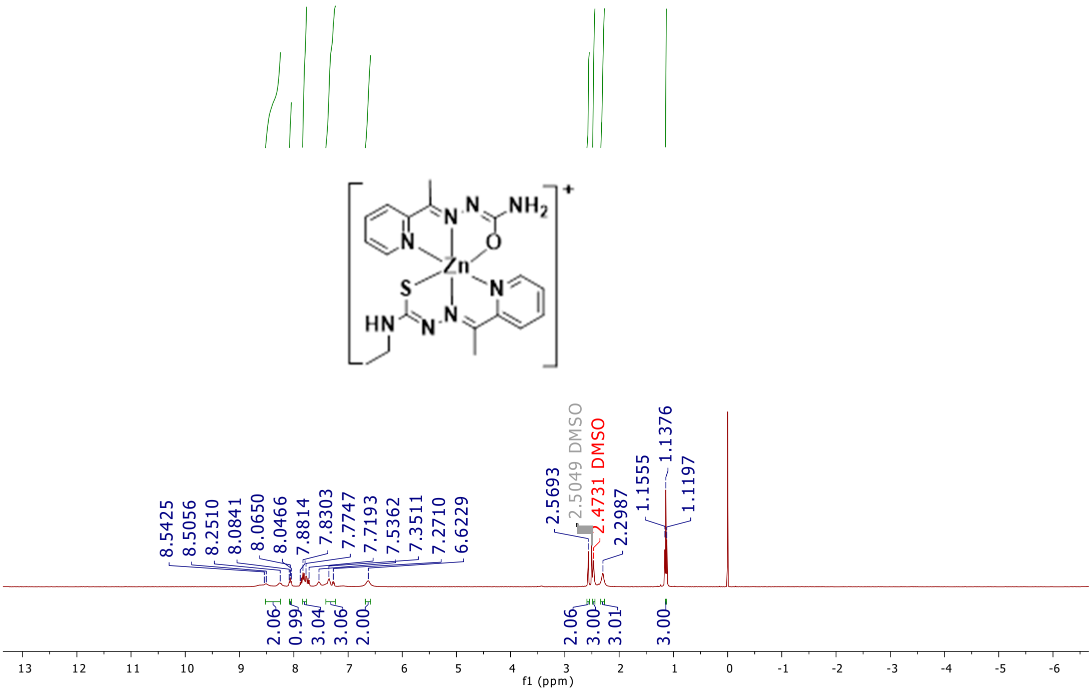

Figure S6: COSY contour map (δ, DMSO, 400 MHz) of Complex **3**.

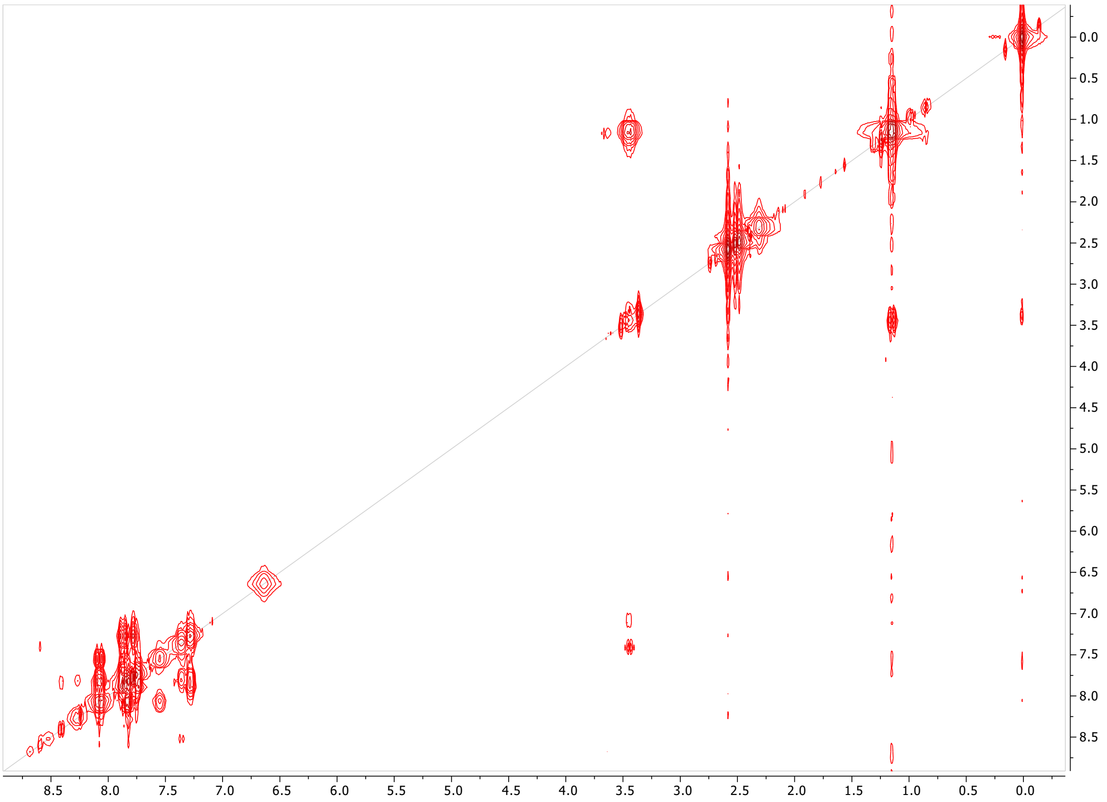

Figure S7: ^{1}H NMR spectrum (400 MHz, DMSO) of Complex **4**.

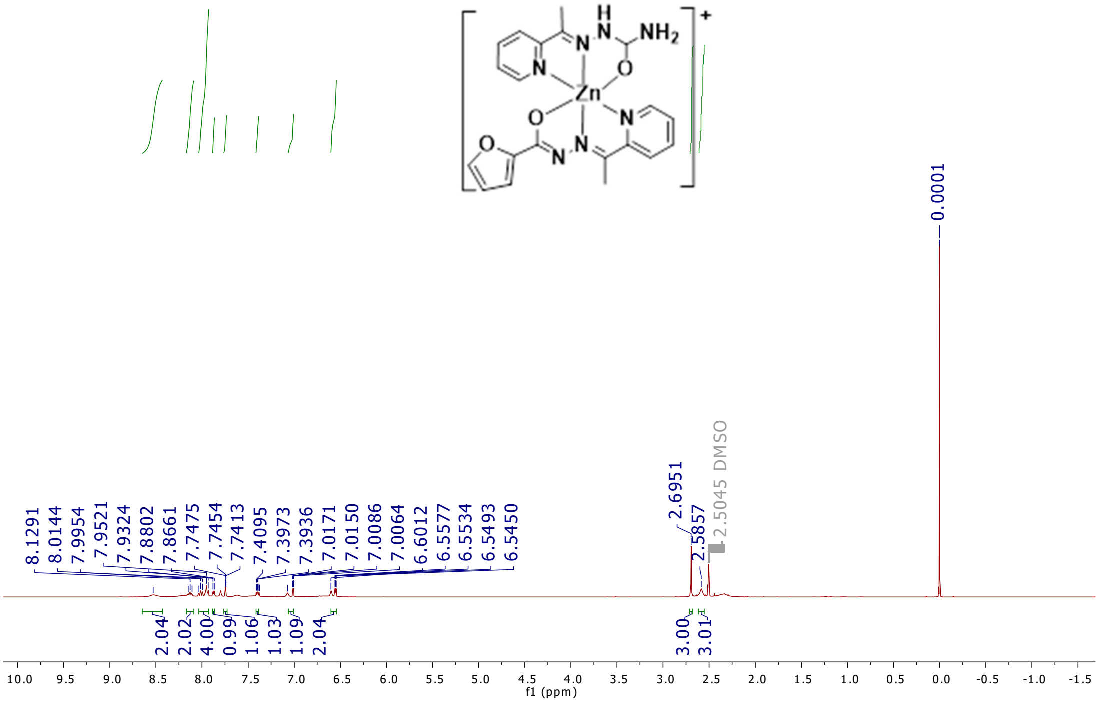

Figure S8: COSY contour map (δ, DMSO, 400 MHz) of Complex **4**.

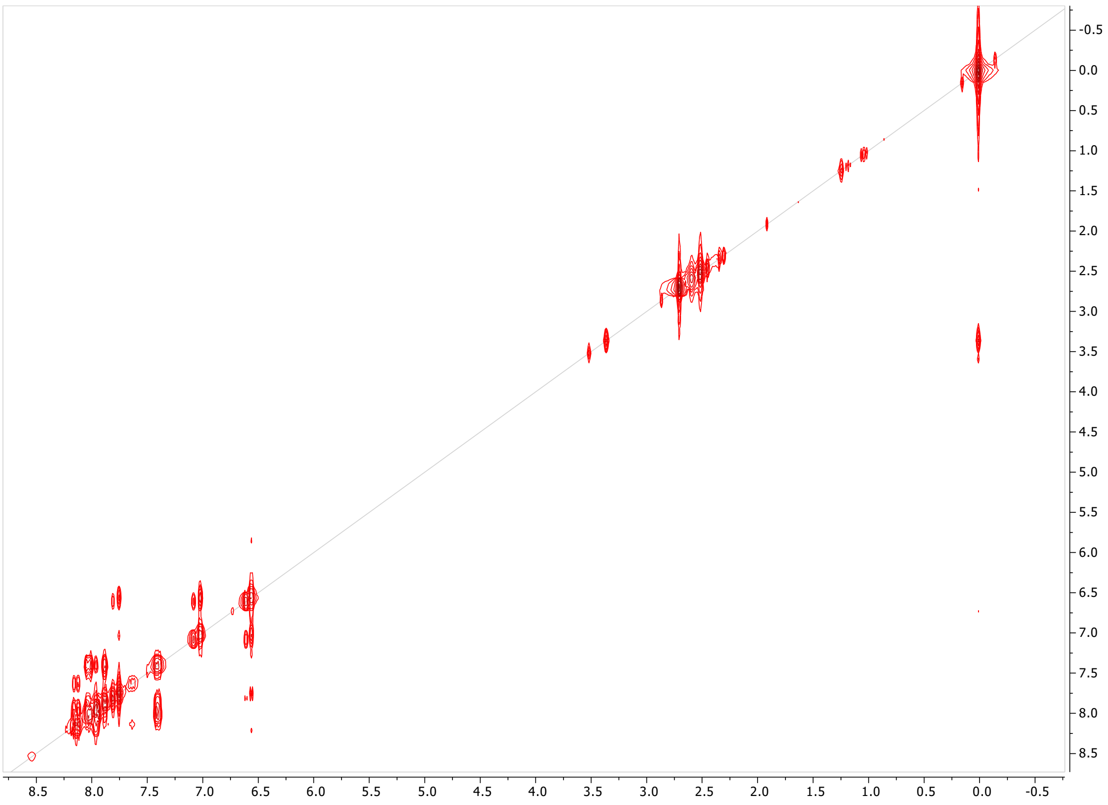

Table S1. Selected bond lengths (Å) and angles (°) for complex **3**.

Bond lengths (Å)		Bond angles (°)	
Zn–S(1)	2.375(2)	S(1)–Zn–O(1)	100.25(13)
Zn–O(1)	2.245(5)	S(1)–Zn–N(1)	154.99(15)
Zn–N(1)	2.164(5)	O(1)–Zn–N(1)	87.47(19)
Zn–N(2)	2.099(5)	N(1)–Zn–N(5)	89.9(2)
Zn–N(5)	2.222(5)	S(1)–Zn–N(6)	111.41(15)
Zn–N(6)	2.145(5)	N(2)–Zn–N(6)	163.58(19)
S(1)–C(8)	1.732(8)	C(8)–S(1)–Zn	95.7(3)
O(1)–C(18)	1.214(8)	C(11)–S(11)–Zn	114.0(4)

Figure S9: Structural packing of Complex **3** in the *b* axis direction. Dashed lines blue and green indicates C–H···O hydrogen bond and C–H···π interaction, respectively.

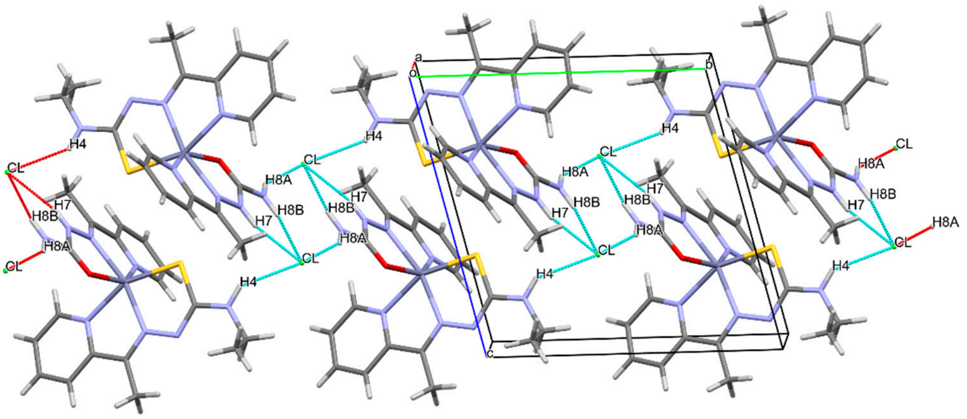

Table S2. Hydrogen–bond geometry (Å, °) in the crystal structure of **3**.

Donor–H···Acceptor	d(D–H)	d(H···A)	d(D···A)	<(DHA)
C7–H7A···N3	0.98	2.35	2.769(9)	105
N8–H8B···Cl	0.88	2.47	3.285(6)	154
N7–H7···Cl	0.88	2.32	3.146(6)	156
N4–H4···Cl^{i}	0.88	2.63	3.354(6)	140
N8–H8A···Cl^{ii}	0.88	2.51	3.290(7)	148
C1–H1···π1	0.95	2.72	3.183(8)	111
C9–H9A···π2	0.99	2.99	3.681(9)	128
C9–H9B···π3^{iii}	0.99	2.92	3.668(9)	133

Symmetry codes: (i) 1–x, –y, 1–z; (ii) 1–x, 1–y, 1–z; (iii) 1–x, –y, –z. π1 is the centroid of ring Zn–N5–C15–C16–N6, π2 is the centroid of ring N1–C1–C2–C3–C4–C5 and π3 is the centroid of ring Zn–N1–C5–C6–N2.

Figure S10: Cytotoxicity of ligands of mixed Zn(II) complexes in MCF7 (ER-positive breast cancer), MDA-MB-453 (HER2-positive breast cancer) and MDA-MB-231 (triple-negative breast cancer) cell lines. The treatment was carried out for 48 hours. (a) Hatc – Et. (B) Hhz. (c) Hhsc. (d) Hatc - Ch. (e) $ZnCl_2$. The experiments were performed in triplicate and the results are expressed as mean ± standard deviation. Significance was calculated by ANOVA and Tukey's post-hoc test. Letters represent significance ($p < 0.05$) between cell lines. a) Control x MCF7, b) Control x MDA-MB-453, c) Control x MDA-MB-231, d) MCF7 x MDA-MB-453, e) MCF7 x MDA-MB-231 and f) MDA-MB -453 x MDA-MB-231.

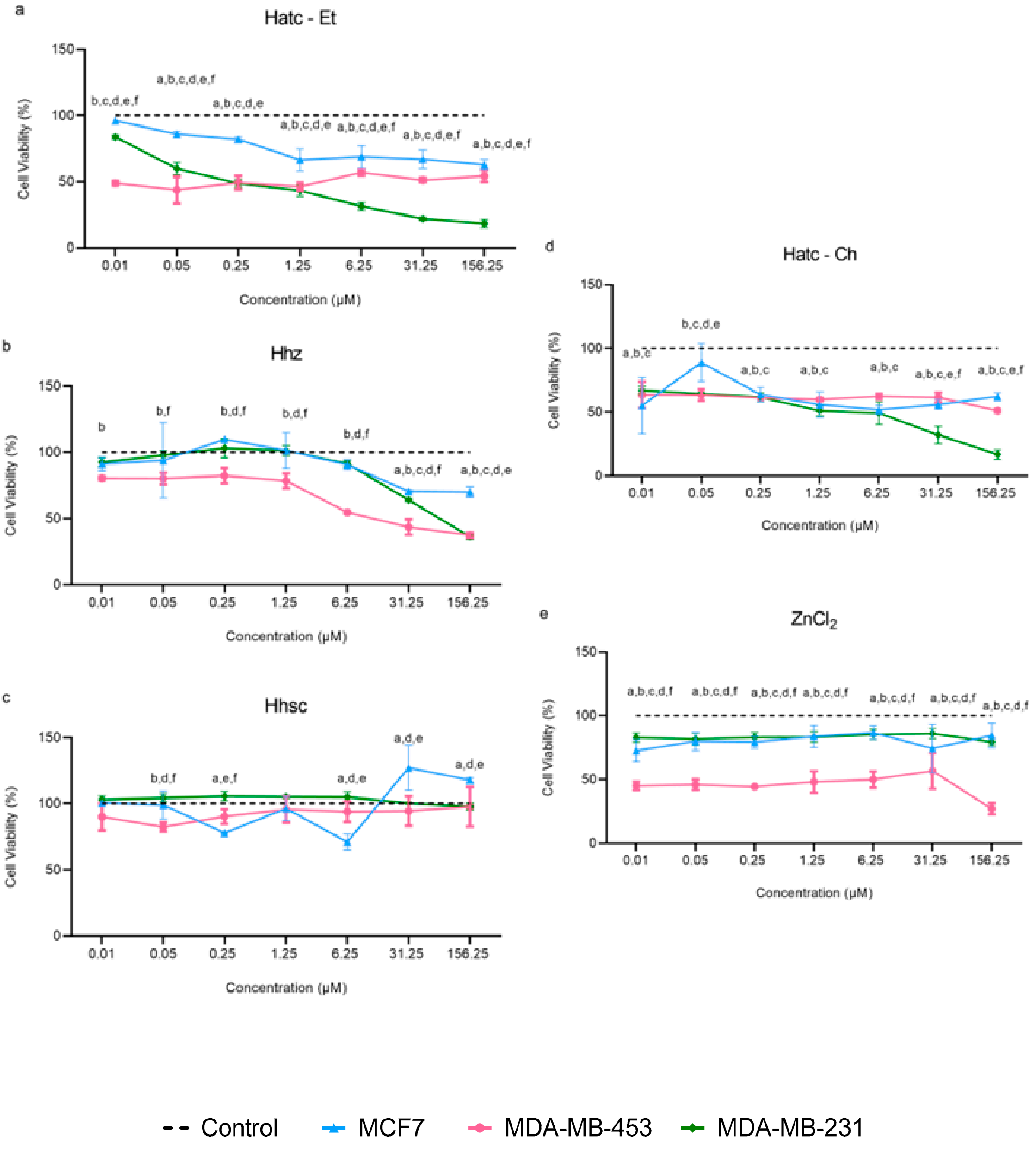

Table S3. Crystallographic data for **3**.

Empirical formula	$C_{18}H_{23}ClN_8OSZn$
Formula weight	500.32
T (K)	203(2)
Wavelength (Å)	0.71073
Crystal system	Triclinic
Space group	*P*-1
Unit cell dimensions	*a* = 9.1195(10) Å α = 72.519(7)°<br>*b* = 11.4189(10) Å β = 70.408(7)°<br>*c* = 11.8714(12) Å γ = 76.005(7)°
V (Å^3)	1097.08(19)
Z	2
ρ_{calc} (mg m^{-3})	1.515
μ (mm^{-1})	1.364
F(000)	516
θ_{min} / θ_{max} (°)	1.88 to 26.47°.
Index ranges	-11<=h<=8,<br>-14<=k<=14,<br>-14<=l<=14
Reflections collected	9833
Independent reflections	4405 [R(int) = 0.0647]
Absorption correction	Multi-scan
T_{max} / T_{min}	0.7452 / 0.6869
Data / restraints / param.	4405 / 0 / 274
GooF	0.971
Final R indices [I>2σ(I)]	R_1 = 0.0701, wR_2 = 0.1606
R indices (all data)	R_1 = 0.1678, wR_2 = 0.2091
Max/min. in Δϱ map (e A^{-3})	0.87 / -0.65
